# Methods for Estimating Wet Bulb Globe Temperature From Remote and Low‐Cost Data: A Comparative Study in Central Alabama

**DOI:** 10.1029/2019GH000231

**Published:** 2020-05-21

**Authors:** Anabel W. Carter, Benjamin F. Zaitchik, Julia M. Gohlke, Suwei Wang, Molly B. Richardson

**Affiliations:** ^1^ Department of Earth and Planetary Sciences Johns Hopkins University Baltimore MD USA; ^2^ Department of Population Health Sciences Virginia Polytechnic Institute and State University Blacksburg VA USA; ^3^ Division of Preventive Medicine University of Alabama at Birmingham Birmingham AL USA

**Keywords:** heat stress, health, WBGT

## Abstract

Heat stress is a significant health concern that can lead to illness, injury, and mortality. The wet bulb globe temperature (WBGT) index is one method for monitoring environmental heat risk. Generally, WBGT is estimated using a heat stress monitor that includes sensors capable of measuring ambient, wet bulb, and black globe temperature, and these measurements are combined to calculate WBGT. However, this method can be expensive, time consuming, and requires careful attention to ensure accurate and repeatable data. Therefore, researchers have attempted to use standard meteorological measurements, using single data sources as an input (e.g., weather stations) to calculate WBGT. Building on these efforts, we apply data from a variety of sources to calculate WBGT, understand the accuracy of our estimated equation, and compare the performance of different sources of input data. To do this, WBGT measurements were collected from Kestrel 5400 Heat Stress Trackers installed in three locations in Alabama. Data were also drawn from local weather stations, North American Land Data Assimilation System (NLDAS), and low cost iButton hygrometers. We applied previously published equations for estimating natural wet bulb temperature, globe temperature, and WBGT to these diverse data sources. Correlation results showed that WBGT estimates derived from all proxy data sources—weather station, weather station/iButton, NLDAS, NLDAS/iButton—were statistically indistinguishable from each other, or from the Kestrel measurements, at two of the three sites. However, at the same two sites, the addition of iButtons significantly reduced root mean square error and bias compared to other methods.

## Key Points

- A variety of proxy methods offered reasonable estimates of wet bulb globe temperature (WBGT) in downtown and suburban Birmingham, AL
- Estimating WBGT from easily accessible measurements could be a powerful tool for studies and interventions related to heat stress

## 1 Introduction

Heat stress is a significant health concern that can lead to illness, injury, and mortality. It can be estimated with a variety of metrics, including absolute or relative thresholds of air temperature, indices that account for temperature and humidity, and apparent temperature estimates that additionally account for radiation and winds (Smith et al., 2013). The wet bulb globe temperature (WBGT) index is a method for monitoring heat stress that is frequently used for setting safe activity standards at workplaces and in athletic and military training facilities. Outdoor WBGT is an estimate of heat stress in direct sunlight and accounts for temperature, humidity, wind speed, sun angle, and solar radiation (SR) in an environment (US Department of Commerce & NOAA, 2019). It is the weighted sum of natural wet bulb (T_nwb_), globe (T_g_), and ambient temperature (T_a_): Each input can be measured directly, while WBGT is a calculated parameter. T_nwb_ is the temperature recorded when a thermometer is covered with a wetted wick, and compared to T_a_, is an indicator of humidity. T_g_ is a measure of radiant temperature and direct measurement requires use of a copper globe painted black, with a thermometer in the center (Dimiceli et al., 2013). WBGT was first developed in the 1950s to help control heat illness during training at military camps. If the WBGT index reached a certain value, training was halted for a given amount of time (Budd, 2008). Today, WBGT is monitored to understand heat stress in a variety of locations, such as offices/work environments, cities, homes, etc. to determine how to proceed in hot environments (Budd, 2008; Hyatt et al., 2010). WBGT is also used to study climate change in many regions of the world (Lemke & Kjellstrom, 2012; Willett & Sherwood, 2012).

Generally, a heat stress monitor is used to measure WBGT at specific locations. These instruments have specialized sensors, such as a wet bulb thermometer and black globe thermometer (Budd, 2008). They are useful because of their accuracy and ability to monitor WBGT at different time intervals, down to a time scale of minutes when necessary. However, it is difficult to measure WBGT in this way because the method is expensive and time consuming. For example, QUESTemp devices are priced around $3,000 per unit, and multiple could be needed for one study (Cooper et al., 2017). Also, data collection becomes time consuming because these measurements require careful attention to ensure accurate and repeatable data (Dimiceli et al., 2013; Liljegren et al., 2008). There are also issues with data collection because of nonstandard instrumentation and unsatisfactory calibration (Budd, 2008; d'Ambrosio Alfano et al., 2014).

For these reasons, researchers have attempted to use standard meteorological measurements to calculate WBGT. A comparison of some of these methods can be found in Lemke and Kjellstrom (2012). For example, Hunter and Minyard (1999) generated a WBGT equation from a regression of T_nwb_ that uses local meteorological measurements. One highly cited method is by Liljegren et al. (2008), who developed a model independent of location and derived from mass and energy balance equations (this method is now used by the National Weather Service). In their review, Lemke and Kjellstrom (2012) highlighted the fact that each method only used one type of data source as an input (e.g., weather station [WS]). The source of meteorological data could be quite important for WBGT applications, in part because WBGT is highly sensitive to microclimate. Variations in radiation due to degree of site shading, in windspeed due to local surface roughness, in humidity and air temperature due to wetness of the surrounding area, and a host of other factors can modify a local monitoring environment in ways that affect WBGT estimates.

Recognizing these challenges, this study uses standard data from a variety of sources to calculate WBGT, understand the accuracy of our estimated equation, and select the best data source for estimating WBGT. To do this, we used WBGT data collected from Kestrel 5400 Heat Stress Trackers installed in three separate locations in Alabama. Kestrel monitors were selected for analysis because they were lower priced than some instruments (although still around $500 each). The experiment was conducted for an extended period of time so we did not want to risk having expensive instruments in exposed, unguarded locations. The study was motivated by the Environmental Health for Alabama Communities (ENACT) project, which had a focus on improved monitoring of heat exposure patterns across rural and urban communities in Alabama.

## 2 Materials and Methods

### 2.1 Data Collection

**FIGURE 1:**
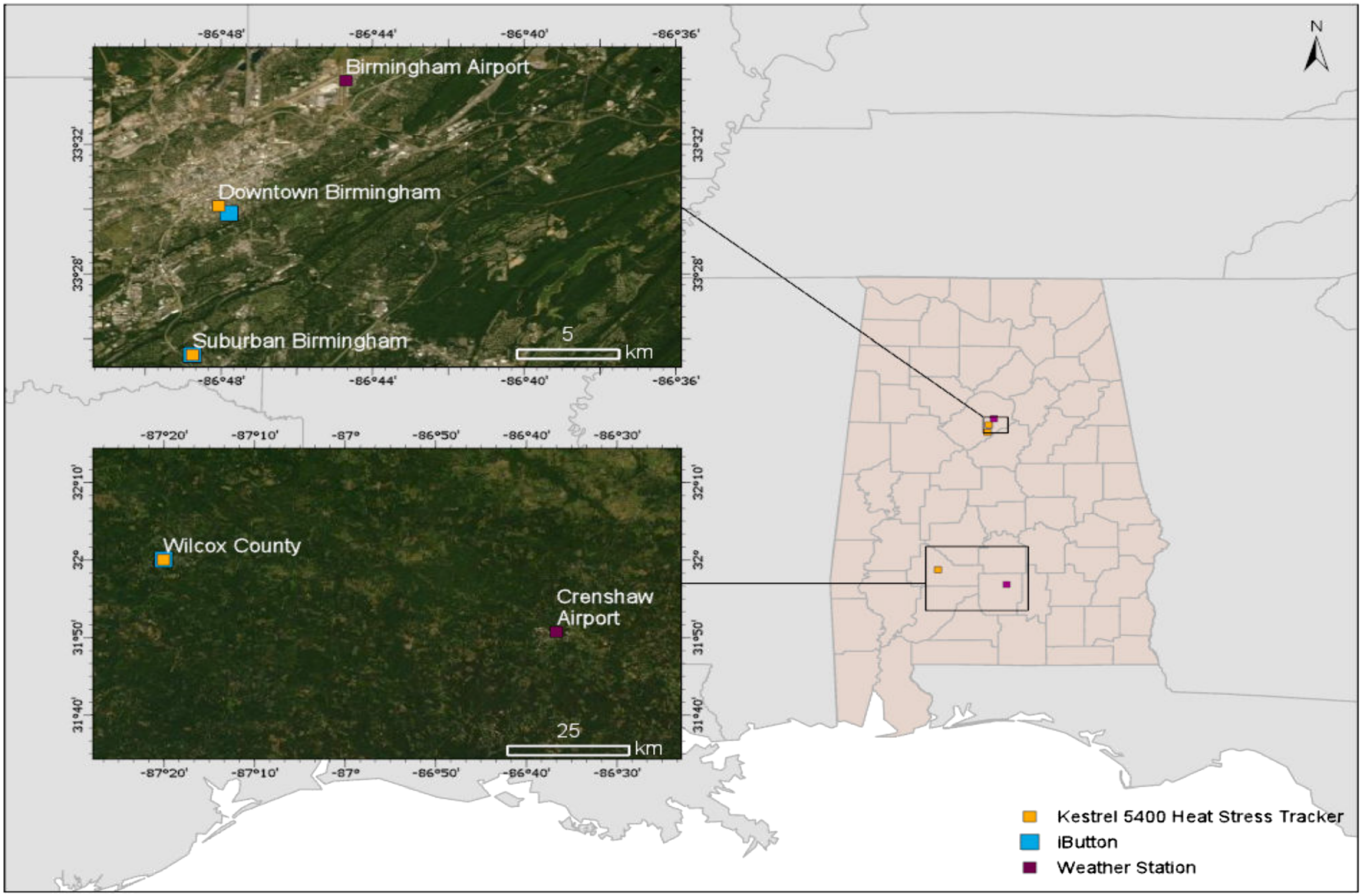
Map of Kestrel monitoring sites, weather stations, and iButtons used in Alabama. Distances between downtown Birmingham data sources are as follows: Kestrel monitor ➔ Birmingham Airport 8.8 km, Kestrel monitor ➔ iButton 0.6 km. Distances between suburban Birmingham data sources are as follows: Kestrel monitor ➔ Birmingham Airport 16.8 km, Kestrel monitor ➔ iButton 0.02 km. Distances between Wilcox County data sources are as follows: Kestrel monitor ➔ Crenshaw Airport 70.5 km, Kestrel monitor ➔ iButton 0.09 km.

Data were collected from three locations in Alabama, which we refer to as the Wilcox County, downtown Birmingham, and suburban Birmingham sites (Figure 1). Two Kestrel 5400 Heat Stress Trackers (*Kestrel monitors*) were installed in Alabama during the spring and summer of 2017. One monitor was placed in Wilcox County (32.0001°N, −87.3343°W) from 5/23 to 6/22. This device was later moved to downtown Birmingham (33.5017°N, −86.8011°W) from 6/23 to 10/20. The second monitor was placed in suburban Birmingham (33.4251°N, −86.8126°W) from 5/23 to 10/20 with a gap in data from 8/14 to 9/7. We must note that the Kestrel 5400 Heat Stress Trackers are not direct WBGT thermometers. The monitors measured T_g_ from a 1 inch globe and used this to calculate T_g_ for a 6 inch globe. The monitors also measured standard meteorological measurements and calculated T_nwb_ and WBGT from the provided measurements. We note that because the Kestrel instrument relies on these scaling relationships (i.e., black globe thermometer is smaller than the standard) and calculations, it does not provide a direct measurement of either T_g_ or T_nwb_. The WBGT estimates derived from Kestrel “measurements,” then, are really a hybrid of measurements and calculations; the instrument includes a 1 inch globe and other measurement capabilities not standard on low‐cost temperature monitoring systems, but it still relies on calculations.

In this study, we treat the Kestrel reading as a pure measurement because it is marketed and used as a commercial grade WBGT monitor, and because it is a reasonable price‐compromise for extended field studies—it is more expensive than a temperature monitor, but not as expensive as the primarily indoor, short‐duration measurement WBGT units used in workplace exposure compliance enforcement (e.g., Quest WBGT meter; NIOSH, 2017). We note, however, that in this regard, the Kestrel measurement is subject to its own limitations. Indeed, we observed occasional erratic measurement behavior for some sunny, low wind days that might be a product of Kestrel measurement errors propagating through the instrument's WBGT calculation. These occasional erratic measurements did not have a significant impact on our results, but we note that they could be a particular concern for direct application of the instrument to heat stress monitoring: sunny, low wind periods are exactly the conditions when heat stress is most likely.

iButton thermometer‐hygrometers—small, portable microchips for gathering data—were installed throughout Birmingham and Wilcox County, Alabama in 2017 to collect relative humidity (RH) and temperature measurements. Forty‐two Maxim Integrated Products, Inc. iButtons (Model DS1923 Hygrochron) were used for analysis. Three iButtons were selected for individual analysis based on their proximity to each Kestrel monitor of interest: downtown Birmingham #37 (33.4979°N, −86.7965°W), suburban Birmingham #39 (33.4252°N, −86.8128°W), and Wilcox County #36 (32.0018°N, −87.3344°W). To protect the iButtons from direct sun exposure, all were installed in shaded areas and equipped with naturally aspirated high reflectivity radiation shields, as described in previous studies (Scott et al., 2017; Scott et al., 2017).

Climate Data Online (CDO), from NOAA's National Climate Data Center, was used to access hourly meteorological measurements from local WSs (Arguez et al., 2010). WSs were selected based on proximity to each Kestrel monitor and on the amount of data they provided. The Birmingham‐Shuttlesworth International Airport (*Birmingham Airport*) WS (33.566°N, −86.745°W) was chosen for downtown and suburban Birmingham. The Mac Crenshaw Memorial Airport (*Crenshaw Airport*) WS (31.846°N, −86.611°W) was selected for Wilcox County. Although Crenshaw Airport was not the closest WS to our Wilcox County monitoring site, it was selected because it had significantly more observations than closer stations (i.e., included dew point temperature).

The NASA Giovanni online data access tool was used to extract hourly time series of North American Land Data Assimilation System (NLDAS) data based on the coordinates from the Kestrel monitors, WSs, and iButtons (Acker & Leptoukh, 2007). NLDAS offers estimates of 2 m height gridded meteorological variables at 12.5 km resolution derived from North American Regional Reanalysis fields that have been downscaled and adjusted using multiple observational data sets (Xia et al., 2012). The following NLDAS meteorological data were downloaded from NASA Giovanni: surface incident shortwave radiation, air temperature, specific humidity, surface pressure, zonal wind, meridional wind.

### 2.2 Data Processing

#### 2.2.1 Dates and Times

All hourly data were adjusted to the CDT (UTC‐5) time zone. Some data sources had missing hours and/or data. For each source, the number of hours in the time period and number of hours in which full data was available is presented, respectively. Kestrel monitors: downtown Birmingham (*n* = 2,871), suburban Birmingham (*n* = 3,034, *n* = 3,005), Wilcox County (*n* = 716).

WSs: WS data that did not correspond with the designated hourly time interval, based on the majority of data, were removed (e.g., 00:59 was removed if majority were 00:56). Birmingham Airport (*n* = 4,411, *n* = 4,407), Crenshaw Airport (n = 4,411, *n* = 4,376). Downtown Birmingham iButton/Birmingham Airport (*n* = 3,940, *n* = 3,936), suburban Birmingham iButton/Birmingham Airport (*n* = 3,938, *n* = 3,934), Wilcox County iButton/Crenshaw Airport (*n* = 3,940, *n* = 3,909). Substituted NLDAS SR data were downloaded based on the WS location of interest.

NLDAS: The number of hours of data from NLDAS was the same in all three locations (*n* = 4,416). These numbers changed slightly with the addition of iButtons: downtown Birmingham iButton/NLDAS (*n* = 3,942), suburban Birmingham iButton/NLDAS (*n* = 4,411), Wilcox County iButton/NLDAS (*n* = 3,942). For the iButton/NLDAS analysis, data with RH >100% were (1) rounded to 100% if there were no large changes in temperature observed at the time of the reading or (2) removed if temperature and humidity changed dramatically and RH rose rapidly above 100% at the time of the observation, as these measurements were interpreted as errors related to condensation on the sensor or other nonstandard processes. No measurements were removed for the individual site analysis. However, for the gridded NLDAS map, five data entries were removed (0.02%) for the month of August.

#### 2.2.2 Meteorological Variables

For analysis, WS data were converted to match measurement units from Kestrel monitors: wind speed was converted to m/s, and all temperatures were changed to degrees Celsius. NLDAS data were also converted for analysis: Temperature was changed from degrees Kelvin to degrees Celsius, and 2 m wind speed was estimated from 10 m zonal and meridional wind speeds. The adjustment of wind speed to 2 m was based on an equation from the FAO Irrigation and Drainage Paper No.56 (Allen et al., 1998) for short grass surfaces:

$$U_{2}=U_{z}\frac{4.87}{\ln\left(67.8z-5.42\right)}.$$

*U*_2_ = wind speed 2 m above ground surface (m/s);

*U*_*z*_ = measured wind speed z m above ground surface (m/s);

*z* = height of measurement above ground surface (m).

Also, surface pressure was converted to millibars and specific humidity to g/kg. RH was calculated from specific humidity, surface pressure, and T_a_. The following equation was used for calculating RH:

$$\mathrm{RH}=\frac{W}{W_{s}*100}.$$

Saturated mixing ratio: $W_{s}=621.97*\frac{\;e_{s}}{P_{\mathrm{sta}}-e_{s}}$. Saturation vapor pressure: $e_{s}=0.6107*\exp\left(\frac{17.27*T_{a}}{T_{a}+237.3}\right).$ Instead of calculating actual mixing ratio (W), we used specific humidity and calculated RH by deriving W_s_ from specific humidity.

### 2.3 WBGT Calculations

#### 2.3.1 Wet Bulb Globe Temperature

Standard meteorological data collected from each site were used to compute WBGT. Outdoor WBGT is calculated as the weighted sum of natural wet bulb temperature (T_nwb_), globe temperature (T_g_), and dry bulb/ambient temperature (T_a_):

$$\text{WBGT}=0.7T_{\mathrm{nwb}}+0.2T_{g}+0.1T_{a}.$$

In order to calculate WBGT from standard measurements, it is necessary to estimate T_nwb_ and T_g_. We used empirically derived equations for analysis. For each site—downtown Birmingham, suburban Birmingham, and Wilcox County—the following data sources were used to generate WBGT: (1) standard meteorological measurements from a Kestrel monitor, (2) local WS, (3) local WS supplemented with iButton data to capture site conditions, (4) NLDAS, and (5) NLDAS supplemented with iButton data. In all cases, SR estimates were drawn from NLDAS, as none of the *in situ* data sources provided that measurement.

#### 2.3.2 Natural Wet Bulb Temperature (T_nwb_)

Equations 1, 2, and 3 of Bernard (1999) were used to estimate natural wet bulb temperature (T_nwb_) from psychrometric wet bulb temperature (T_pwb_). The equations were recommended and used by the Kestrel monitor manufacturers (Naughton, 2016). Equation 1 was used to calculate T_nwb_ if *T*_*g*_ − *T*_*a*_ < 4:

$$T_{\mathrm{nwb}}=T_{a}-C\left(T_{a}-T_{\mathrm{pwb}}\right),$$

$$V=\text{wind speed}\;\left(\frac{m}{s}\right).$$

$$C=0.85\;\text{for}\;V<0.03\frac{m}{s},$$

$$C=1.0\;\text{for}\;V>3.0\frac{m}{s},$$

$$C=0.96+0.069\log_{10}V\;\text{for}\;0.03\leq V\leq 3.0\;\frac{m}{s}.$$

If *T*_*g*_ − *T*_*a*_ ≥ 4, equation 2 was used to incorporate the effect of radiant heat:

$$T_{\mathrm{nwb}}=T_{\mathrm{pwb}}+0.25\left(T_{g}-T_{a}\right)+e,$$

$$e=1.1\;\text{for}\;V<0.1\;\frac{m}{s},$$

$$e=-0.1\;\text{for}\;V=1.0\;\frac{m}{s},$$

$$e=0.10/V^{1.1}-0.2\;\text{for}\;0.1\leq V\leq 1.0\frac{m}{s}.$$

The equation for T_pwb_ (equation 3) is as follows:

$$T_{\mathrm{pwb}}=0.376+5.79e_{a}+\left(0.388-0.0465e_{a}\right)T_{a}.$$

Ambient vapor pressure (e_a_) was computed as part of the calculation for T_nwb_. Bernard (1999) provides an equation for e_a_:

$$e_{a}=\left(\frac{\mathrm{RH}}{100}\right)*\left(0.6107\exp\left[\frac{17.27T_{a}}{T_{a}+237.3}\right]\right).$$

However, one input is RH, which is not provided by all data sources (i.e., WSs). Therefore, when necessary, e_a_ was calculated using dew point temperature (T_d_) (Campbell & Norman, 1998):

$$e_{a}\left(T_{d}\right)=a\;\exp\left(\frac{bT_{d}}{T_{d}+c}\right).$$

The values of a, b, and c come from Bernard (1999): a = 0.6107, b = 17.27, and c = 237.3.

#### 2.3.3 Globe Temperature (T_g_)

To calculate T_g_, we followed a method similar to Hajizadeh et al. (2017) in which a regression was fit based on SR, T_a_, RH, and known T_g_. We generated a single, cross‐site equation for T_g_ based on Kestrel monitor data from all three sites. This process allowed us to apply one equation to all locations. T_a_, RH, and T_g_ data came from the Kestrel monitors to create this equation because it is the only device that generated T_g_. SR data came from NASA Giovanni NLDAS. We used a 30% holdout to generate the T_g_ equation since it was based on data from the same device in which T_g_ was measured. The holdout was generated by withholding the last 30% of the measurement period for each study site. The 30% holdout equation specific to our data is

$$T_{g}=0.009624\left(\mathrm{SR}\right)+1.102\left(T_{a}\right)-0.00404\left(\mathrm{RH}\right)-2.2776.$$

### 2.4 Data Analysis

#### 2.4.1 Predictor Values

At each location, T_a_, wind speed, and RH values were compared to understand how predictor values might change between data sources. We looked at the following relationships when applicable: (1) Kestrel monitor vs. NLDAS, (2) Kestrel monitor vs. WS, (3) Kestrel monitor vs. iButton, and (4) NLDAS vs. WS. *R* ^2^, root mean square error (RMSE), and bias were calculated for all relationships.

#### 2.4.2 Linear Regressions

At each study location, scatter plots were generated for Kestrel reported vs. calculated values of T_g_, T_nwb_, and WBGT to visualize the performance of each equation. Linear regressions were run for the Kestrel reported vs. calculated T_g_, T_nwb_, and WBGT plots, and for the following relationships: instrument reported WBGT vs. estimates based on local WS data, instrument reported WBGT vs. estimates based on local WS and closest iButton data, instrument reported WBGT vs. estimates based solely on NLDAS, and instrument reported WBGT vs. estimates based on NLDAS and closest iButton data. *R* ^2^, RMSE, and bias were calculated for all relationships.

#### 2.4.3 Time Series

Daily average time series are presented for predictor values (wind speed, T_a_, RH) and WBGT variables (T_nwb_, T_g_, WBGT). Time series of predictor values present data from the Kestrel monitors, iButtons, WSs, and NLDAS at each monitoring site. Time series of WBGT variables present instrument reported and estimated data at each monitoring site. We also completed diurnal cycles of each variable in suburban Birmingham on 7 October 2017.

#### 2.4.4 Significance Tests

As there is strong temporal autocorrelation in our data at hourly and daily timescales, we applied an effective sample size correction when performing significance tests. A lag‐1 equation for effective sample size was used: N_eff_ $=N*\frac{1-r_{1}}{1+r_{1}}$, *N* = original sample size and *r*_*1*_ = lag‐1 correlation. Fisher's r to z transformations were run to analyze the relationship between correlation coefficients and help assess each calculation method. To do this, N_eff_ was first calculated for each correlation by averaging N_eff_ for each Kestrel reported and calculated WBGT. The Fisher's r to z transformations were then run using the averaged N_eff_ values to compare each correlation (Fisher, 1915). We also ran one sample t‐tests, $t=\frac{\underline{x}-\mu_{0}}{s/N}$( $\underline{x\;}$= mean, *μ*_0_ = 0, s = standard deviation, N = sample size), for bias and RMSE, where N = N_eff_. Significance tests for bias were run between estimation methods, and between Kestrel on‐board values and proxy methods. T‐tests for RMSE were run between estimation methods. Significance tests for predictor meteorological variables were performed using the entire data record, as these measurements are mutually independent from each other. Evaluation of T_g_, T_nwb_, and WBGT were performed using the 30% holdout, since those estimates depend on equations derived using training data.

#### 2.4.5 Occupational Heat Stress Thresholds

Each of the 42 iButtons was paired with the closest WS (either Birmingham Airport or Crenshaw Airport) and WBGT was calculated. Standard work hours from WS/iButton WBGT estimates were included (9:00–16:00 inclusive) for the month of August 2017 (*n* = 248 for each iButton in Jefferson County, *n* = 246 for each iButton in Wilcox County). The National Institute for Occupational Safety and Health (NIOSH) 2017 thresholds for % work recommended per hour were applied to the hourly data: Continuous work <28°C, 75–100% work (45 to 60 min work/0 to 15 min rest per hour) >28°C and <29°C, 50–75% work (30 to 45 min work/15 to 30 min rest per hour) >29°C and <30°C, 25–50% work (15 to 30 min work/30 to 45 min rest per hour) >30°C and <31.5°C, 0–25% work (0 to 15 min work/45 to 60 min rest per hour) ≥31.5°C. The percentage (%) of work hours that fell within each work‐rest category was calculated for each iButton. The percentages were averaged across all the iButtons in Jefferson County and Wilcox County, respectively. The same thresholds were applied to WSs in Jefferson County (Birmingham Airport, *n* = 248) and Wilcox County (Crenshaw Airport, *n* = 246).

## 3 Results and Discussion

### 3.1 Predictor Values

**FIGURE 2:**
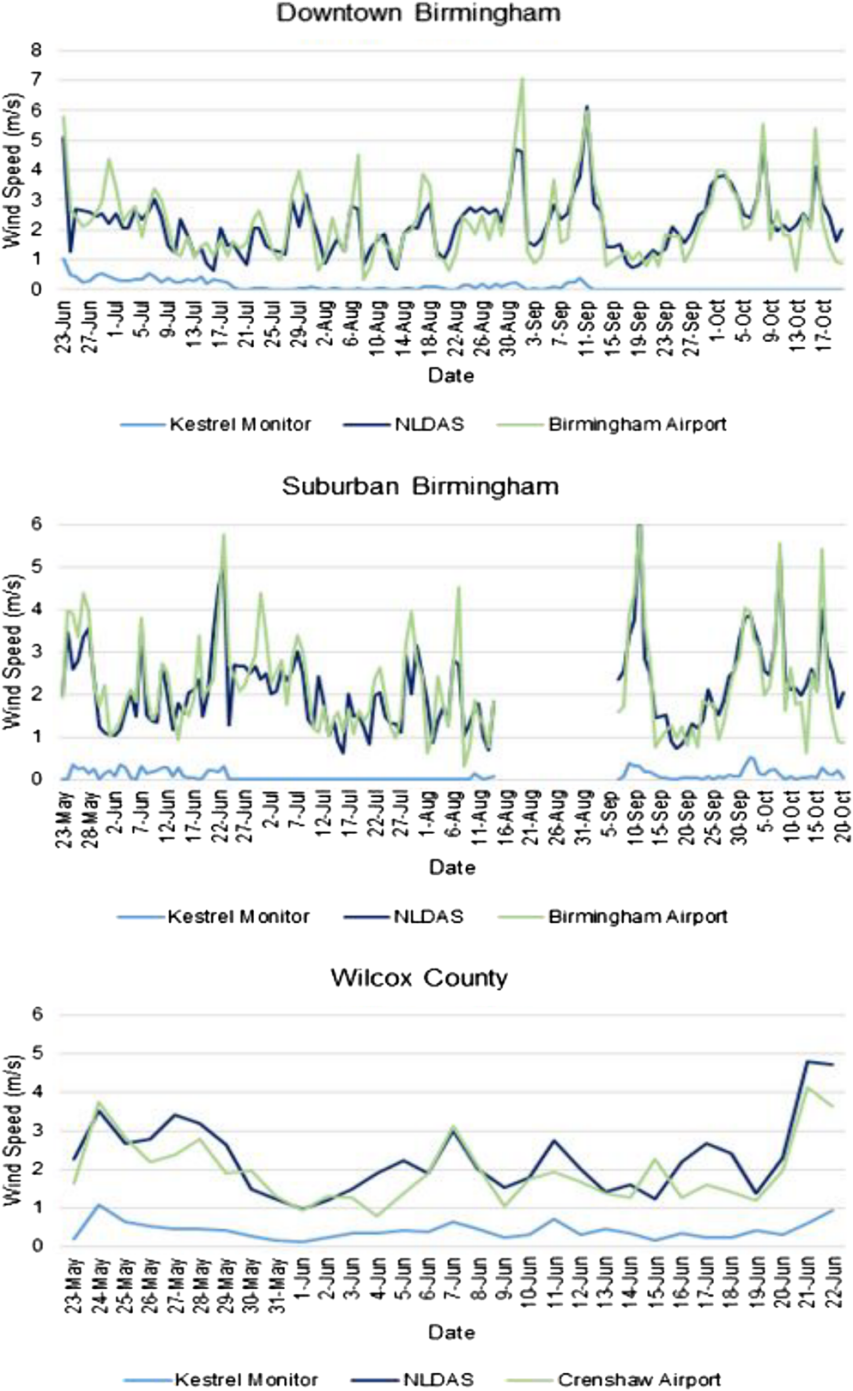
Daily average time series of wind speed at each monitor site.

Wind speed, T_a_, and RH were collected from NLDAS, WSs, Kestrel monitors, while iButtons collected only temperature and humidity. For each data source, time series were constructed, and linear regressions were run to help explain the variation seen in WBGT calculations using different methods. This analysis indicated significant differences (*p* < 0.05) between wind speed measurements taken by the Kestrel monitor and all other methods, *R* ^2^ < 0.60 in all locations (Figure 2, Table 1). The weakest relationship was observed between the Kestrel monitor and NLDAS in downtown Birmingham (*R* ^2^ = 0.05), reflecting the significant impact of urban surface roughness on winds. The low wind speeds measured by the Kestrel monitors across sites are likely due to true differences in microclimates — they were installed in residential and commercial areas that are sheltered from wind relative to an airport or open field WS. If low wind speed is due to location, we expect WBGT to be higher in these environments, all else being equal. However, low wind speed could also be due to limitations in the instruments' sensitivity; it is possible that the monitors were not capable of accurately gathering moderate to low wind speed. This is again relevant to WBGT estimates, as lower wind speed means less airflow and subsequent higher WBGT.

**TABLE 1:** Regression Results for Predictor Values (Wind Speed, T_a_, and RH) in Downtown Birmingham (*n* = 120), Suburban Birmingham (*n* = 128), and Wilcox County (*n* = 31)

Observations	Regression	Downtown Birmingham			Suburban Birmingham			Wilcox County		
		*R* ^2^	RMSE	Bias	*R* ^2^	RMSE	Bias	*R* ^2^	RMSE	Bias
Wind speed (m/s)	Kestrel monitor vs. NLDAS	0.05^a^	2.30	−2.09^*^	0.24^a^	2.25	−2.06^*^	0.53	2.03	−1.87^*^
	Kestrel monitor vs. weather station	0.09^a^	2.44	−2.12^*^	0.19^a^	2.39	−2.13^*^	0.57	1.67	−1.53^*^
	NLDAS vs. weather station	0.73^b^	0.66	−0.03	0.71^b^	0.62	−0.07	0.70	0.61	0.34^*^
Temperature (°C)	Kestrel monitor vs. NLDAS	0.88^a^	1.77	1.24^*^	0.80^a^	1.57	0.29^*^	0.47^a^	1.54	−0.60^*^
	Kestrel monitor vs. weather station	0.92^ab^	1.67	1.38^*^	0.88^b^	1.14	0.36^*^	0.76^a^	0.80	0.21
	Kestrel monitor vs. iButton	0.95^b^	1.10	0.75^*^	0.94^c^	1.00	0.62^*^	0.94^b^	0.39	−0.08
	NLDAS vs. weather station	0.91^a^	1.12	0.13	0.90^b^	1.11	0.07	0.51^a^	1.56	0.81^*^
Relative Humidity (%)	Kestrel monitor vs. NLDAS	0.63^ab^	9.83	2.83^*^	0.52^a^	11.16	5.56^*^	0.50^a^	11.03	7.94^*^
	Kestrel monitor vs. weather station	0.61^ab^	9.02	1.68^*^	0.74^b^	8.31	4.44^*^	0.71^a^	11.80	10.35^*^
	Kestrel monitor vs. iButton	0.71^b^	7.72	2.01^*^	0.90^c^	4.60	−1.01^*^	0.91^b^	3.76	1.70^*^
	NLDAS vs. weather station	0.53^a^	6.40	−1.15^*^	0.54^a^	6.71	−1.11	0.69^a^	5.13	2.41^*^

Note. The * indicates significant difference between measurements (one sample *t*‐test), and letters indicate differences between regressions.

**FIGURE 3:**
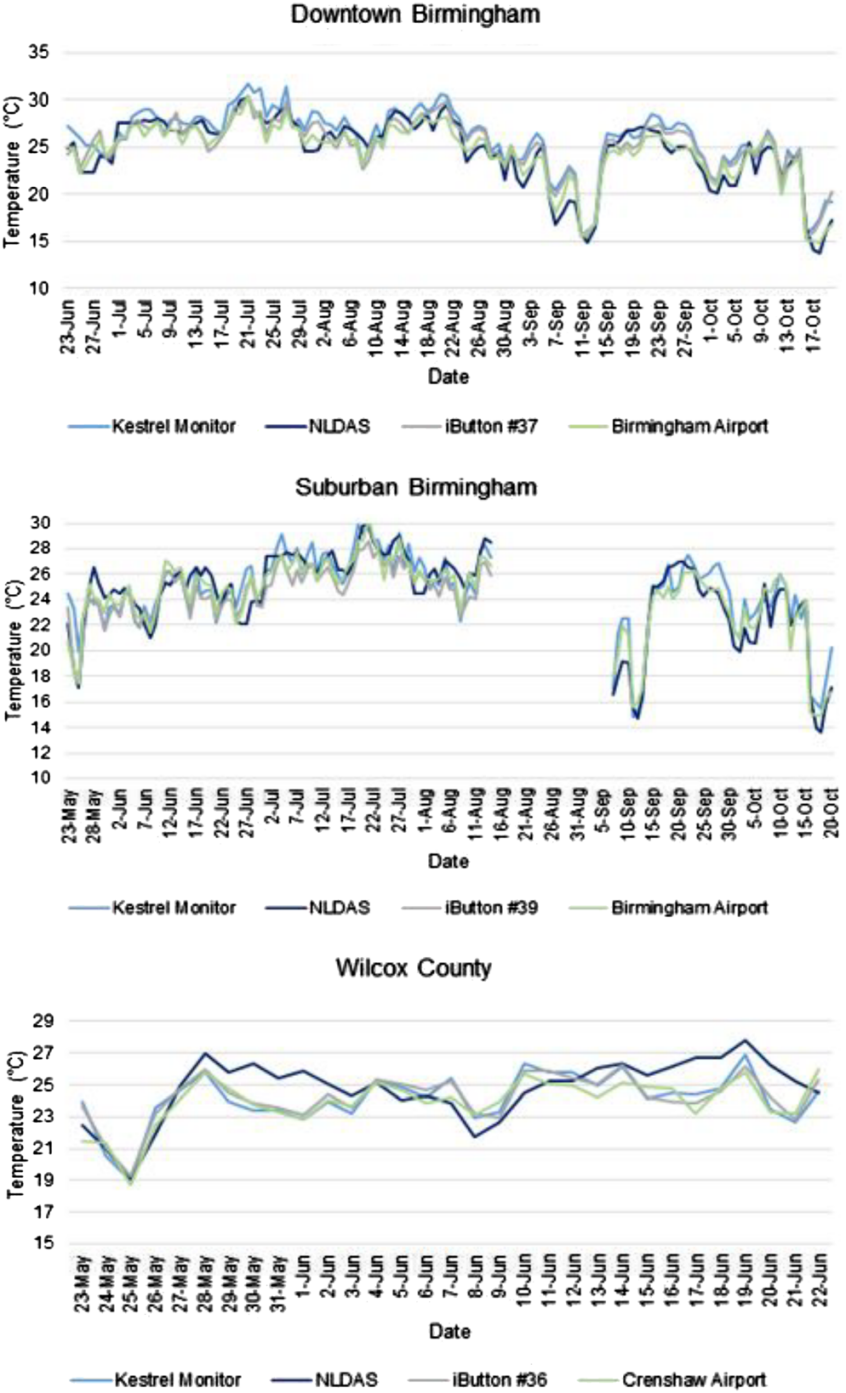
Daily average time series of temperature (T_a_) at each monitor site.

Temperature did not vary as much between instruments. There were some slight differences in temperature for each method, but it was not possible to draw distinctions based on the time series (Figure 3). Linear regression results showed that Kestrel temperatures were similar to all monitoring devices in downtown and suburban Birmingham, *R* ^2^ ≥ 0.80 (Table 1). However, in Wilcox County, there was more variation between devices (0.47 ≤ *R* ^2^ ≤ 0.94). This variation is possibly a result of the short data record and, for some comparisons, distance from the WS. We also observed that the strongest relationships, in each location, were between Kestrel monitors and iButtons. This is likely due to the placement of the iButtons and monitors. In each study site, an iButton was placed near a monitor so their environments were similar.

**FIGURE 4:**
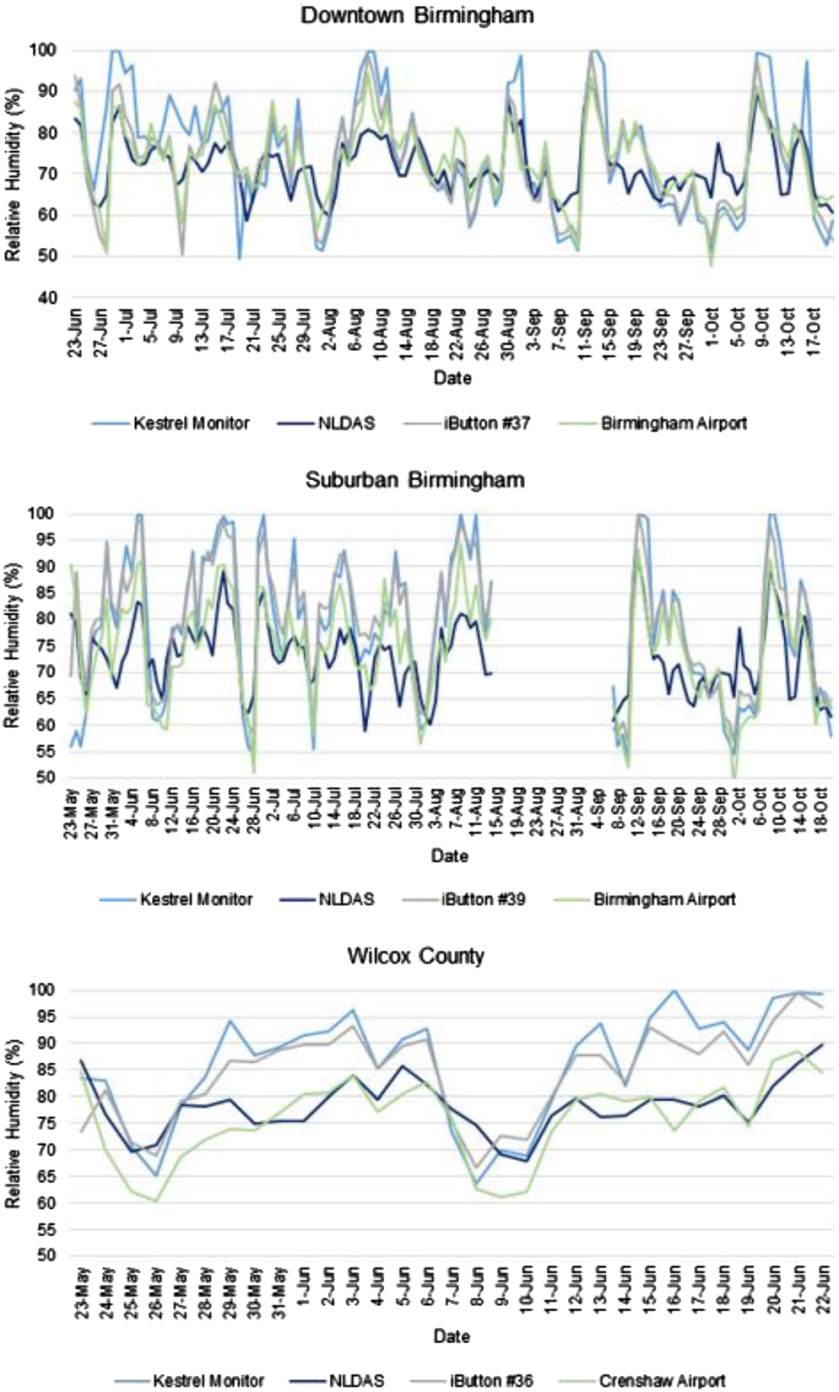
Daily average time series of relative humidity at each monitor site.

The time series also showed some clearer distinctions between measurements for RH (Figure 4). Almost all differences were significant, and we observed relatively weak relationships across all locations (Table 1). This was likely due to the spatial variability of humidity. However, the iButtons and Kestrel monitors followed similar trends and had the strongest relationships, which is supported by high *R* ^2^ values (Table 1). Again, this can possibly be explained by the proximity of the Kestrel monitors and iButtons. As for NLDAS RH, the weak relationships between it and other devices could be due to spatial averaging and the calculation method for RH. NLDAS generally showed lower temporal variability in RH than was reported by *in situ* methods.

### 3.2 T_nwb_, T_g_, and WBGT From Kestrel 5400 Heat Stress Tracker

**FIGURE 5:**
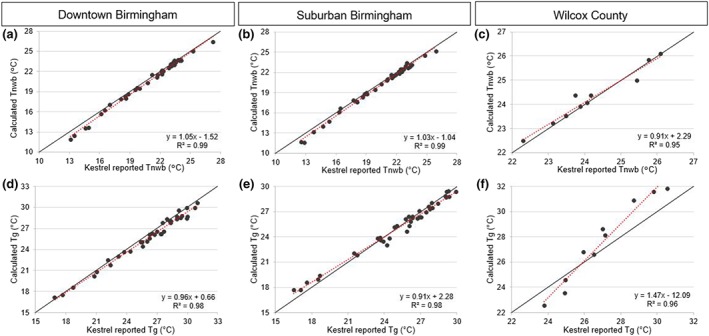
Linear regressions of Kestrel reported T_g_ and T_nwb_ vs. calculated T_g_ and T_nwb_ based on daily averages. Black solid lines represent a 1/1 slope. Downtown Birmingham (*n* = 37), suburban Birmingham (*n* = 38), Wilcox County (*n* = 10).

Linear regressions were run to compare instrument reported values of T_g_ and T_nwb_ to values calculated using the equations selected for this study. Results of the linear regressions showed the ability of our equations to replicate the Kestrel reported WBGT. There were strong relationships between Kestrel reported and calculated daily averaged T_nwb_ values: *R* ^2^ ≥ 0.95 for all locations, with regression coefficients close to 1.0 (Figures 5a–5c). Time series of reported and calculated T_nwb_ values also showed strong agreement at each study location at daily time scales (Figure 7a). We note that this agreement is largely due to the fact that T_nwb_ is a derived parameter for the Kestrel instrument, calculated using a similar set of inputs to those we apply. We used the same equation by Bernard (1999) that the Kestrel products used to calculate T_nwb_. The strong agreement should thus be interpreted as evidence that our choice of T_nwb_ equation supports our estimates of WBGT.

There were also strong relationships between reported and calculated daily averaged T_g_ values in all locations, *R* ^2^ ≥ 0.96 (Figures 5d–5f). However, in Wilcox County, the regression coefficient was 1.47 (Figure 5f). The time series highlights the observed difference in T_g_ between reported and calculated values (Figure 7b). These results may be explained in part by the lack of data for Wilcox County. We used 5 months of data for both downtown and suburban Birmingham, while Wilcox County only had 1 month of measurements. Even fewer data were used for testing the final regression once the 30% holdout was applied. This presents a standard data volume challenge, in that there were fewer data points for Wilcox County. It also meant that Wilcox County was systematically underrepresented when we pooled data to fit a single cross‐site equation. It is difficult to draw conclusions from the Wilcox County results because of this underrepresentation. The data appear to be highly correlated when looking at a few days (Figure 5f), but this could easily change if the data collection period were longer. The ambiguous result suggests that caution should be used when applying T_g_ coefficients derived from one environment to another environment, even within the same climate zone and the same general region. The results also might be an indicator of the limits of using a fairly simple equation, like the approach by Hajizadeh et al. (2017) that we used here. Methods that apply a full energy balance equation, though more data intensive, might be more robust to changes in environment. Since T_g_ is measured and then adjusted by the Kestrel instrument, comparisons with our calculated estimates are fairly independent comparisons of estimates to measurement.

**FIGURE 6:**
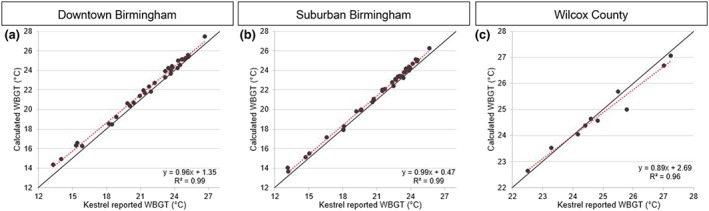
Linear regressions of Kestrel reported WBGT vs. calculated WBGT based on daily averages. Black solid lines represent a 1/1 slope.

**FIGURE 7:**
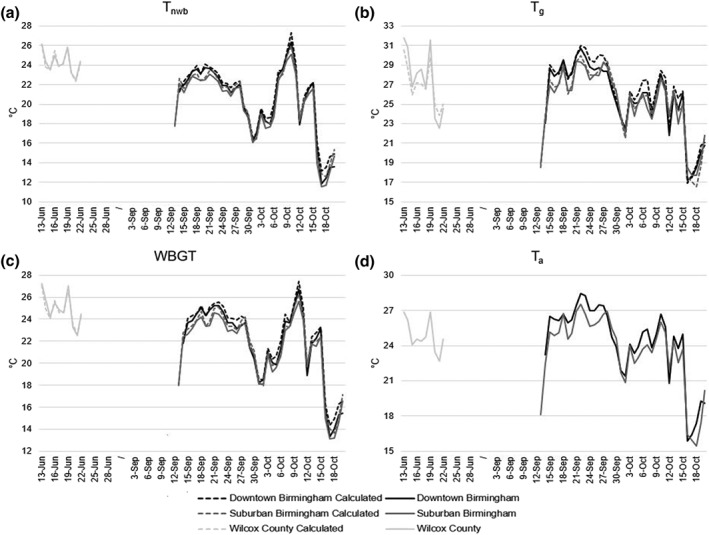
Daily average time series of instrument recorded and calculated values for T_g_, T_nwb_, T_a_, and WBGT for downtown Birmingham, suburban Birmingham, and Wilcox County for the holdout period.

Notwithstanding the performance of our T_g_ estimate at the Wilcox County site, we found strong relationships between known and calculated daily average WBGT values at each location (Figures 6 and 7c). *R* ^2^ ≥ 0.96 for all locations and the regression coefficients were close to 1. The slight differences in WBGT can be explained by variation in T_g_ and T_nwb_ between instrument reported and calculated values. T_nwb_ is 70% of the calculation for WBGT; therefore, small differences in T_nwb_ will reflect lower *R* ^2^ values for WBGT. This is true for T_g_ as well, but to a lesser extent since T_g_ is only 20% of WBGT equation. Overall, the results show that we can estimate the Kestrel instruments' reported daily average WBGT value using on‐board measurements of predictor variables.

The time series shown in Figure 7 present differences between reported and calculated values. They also highlight the variation in heat stress from day to day and some differences between study sites. Most WBGTs in downtown and suburban Birmingham average below 26°C. However, on 10 October 2017, we observe a spike in Kestrel T_nwb_ which is responsible for the very high WBGT that same day.

### 3.3 WBGT Estimates

While it is useful to show that WBGT can be reconstructed using a suite of standard meteorological measurements on‐board the Kestrel instrument, our objective is to evaluate our ability to estimate WBGT using less expensive or existing meteorological measurements and estimates. To do this, we assessed our ability to estimate daily average WBGT using WS, WS/iButton, NLDAS, and NLDAS/iButton data in place of the on‐board Kestrel measurements used when fitting the equations. We then ran linear regressions to compare measured WBGT values from the Kestrel monitors to calculated values from various instruments (Table 2). The regression results for Kestrel reported vs. calculated WBGT were previously presented in Figure 6, and they are included in the table as a point of reference. It is expected that these results will have the strongest *R* ^2^ values. Results show that all proxy estimates, that do not use onboard measurements, under predict WBGT. Based on our results, it is difficult to assess the best instrument for estimating WBGT, because *R* ^2^, regression coefficient, and RMSE values fluctuate. Significance tests show that there is no statistical way to distinguish the correlations in downtown and suburban Birmingham. That said, only considering *R* ^2^ and the regression coefficient, the best instrument for calculating WBGT in each location is the WS. Each location also shares the weakest relationship which is Kestrel reported vs. NLDAS (Table 2). NLDAS is likely the worst proxy for predicting WBGT because it is a spatially averaged data assimilation product that relies heavily on models to generate estimates of surface meteorological conditions.

**TABLE 2:** Linear Regression Results for Kestrel Reported WBGT vs. Various Estimates in Downtown Birmingham, Suburban Birmingham, and Wilcox County

	*R* ^2^	Regression coefficient	RMSE	Bias
**Downtown Birmingham**				
Kestrel reported vs. calculated	0.99	0.96	0.54^a^	−0.44^a*^
Kestrel reported vs. weather station	0.98	0.96	1.32^b^	1.22^b*^
Kestrel reported vs. weather station/iButton	0.97	0.90	0.73^a^	0.32^c^
Kestrel reported vs. NLDAS	0.96	0.95	1.36^b^	1.18^b*^
Kestrel reported vs. NLDAS/iButton	0.97	0.90	0.75^a^	0.42^d*^
**Suburban Birmingham**				
Kestrel reported vs. calculated	0.99	0.99	0.42^ac^	−0.36^a*^
Kestrel reported vs. weather station	0.97	1.02	1.01^b^	0.80^bd*^
Kestrel reported vs. weather station/iButton	0.97	0.95	0.61^a^	0.11^c^
Kestrel reported vs. NLDAS	0.94	1.00	1.23^b^	0.91^b*^
Kestrel reported vs. NLDAS/iButton	0.97	0.94	0.71^c^	0.42^d*^
**Wilcox County**				
Kestrel reported vs. calculated	0.96^a^	0.89	0.31^ab^	0.11^a^
Kestrel reported vs. weather station	0.68^ab^	0.65	0.97^ab^	0.54^a*^
Kestrel reported vs. weather station/iButton	0.36^b^	0.35	1.40^c^	0.82^a^
Kestrel reported vs. NLDAS	0.18^b^	0.20	1.33^bc^	0.33^a^
Kestrel reported vs. NLDAS/iButton	0.39^b^	0.37	1.35^c^	0.76^a^

Note. The * indicates significant difference from Kestrel (one sample t‐test), and letters indicate significant differences between methods.

Due to the proximity of each iButton to the Kestrel monitors, it might be inferred that by adding iButton data to WS or NLDAS measurements, the WBGT prediction would increase accuracy. This assumption is partially supported by the results. In downtown and suburban Birmingham, RMSE and bias values were significantly lower when iButton data were added to WSs or NLDAS (Table 2). Also, in all locations, *R* ^2^ increased when iButton data were added to NLDAS. However, when iButton data were added to the WSs, the *R* ^2^ decreased, and dramatically so in Wilcox County. Also, in most instances, the regression coefficient moved further from one with the addition of the iButtons, perhaps reflecting a different dynamic response of iButton hygrometers relative to the Kestrel monitors and other methods.

It is important to note that all *R* ^2^ values are below 0.80 in Wilcox County (besides Kestrel reported vs. calculated). Therefore, the strongest relationships for Wilcox County are relative measures for relatively poorly performing methods. Weaker correlations are probably due to the smaller sample size for Wilcox County.

### 3.4 Diurnal Cycle and Daily Maximum

**FIGURE 8:**
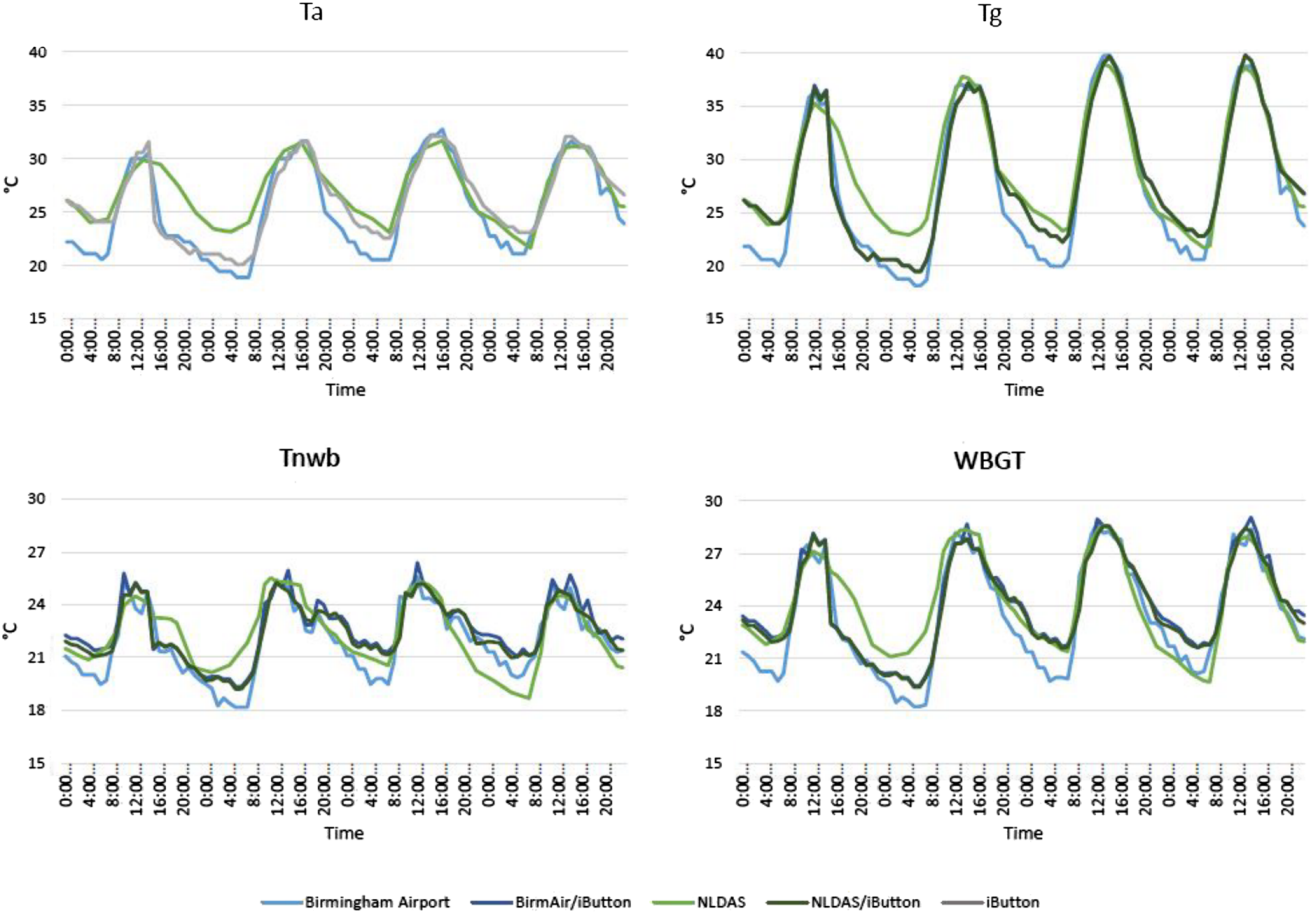
Diurnal cycles of T_a_, T_g_, T_nwb_, and WBGT in downtown Birmingham from 19–22 September 2017.

T_a_, T_g_, T_nwb_, and WBGT estimates and measurements were plotted for a 4‐day period, 19–22 September 2017, at the downtown Birmingham site (Figure 8). The diurnal cycles presented here are not an overall reflection of our estimates of heat stress. Rather, the cycles highlight the behavior of our estimates throughout 24‐hr periods. All estimation methods peaked in the early afternoon. However, there was variation in the magnitude of the diurnal cycle in WBGT and its constituent terms. We observed the most variability during the morning hours. However, during the time period, the three to four hottest, and riskiest, hours of the day appear to be the least variable across methods.

We note that the 4 days selected for Figure 8 are some of the hottest for the study period (temperatures peak around 28°C). It is also notable that NLDAS shows the smoothest diurnal cycle, reflecting the fact that it is a grid scale‐averaged temperature estimate that is largely model derived. There is more variation from hour to hour when iButton data are used in combination with NLDAS.

Based on the previous results, and to better understand variability between methods under the hottest conditions, we compared daily maximum WBGT from each of the proposed proxy estimation methods at each study location during the entire study period (Table 3). Results indicate that the mean daily maximum WBGT estimate provided by different methods can differ by up to 1°C (1.8°F), with the largest difference found for the Downtown Birmingham study site. These differences in mean daily maximum WBGT were not statistically significant (*p* > 0.05), but they might be meaningful under some circumstances. Linear correlation between estimation methods in downtown and suburban Birmingham was very strong. Daily root mean square differences (RMSD) between methods ranged from 0.26–1.58°C across sites and methods. There is a tendency for RMSD to be largest between methods that rely on NLDAS and the *in situ* based methods, but the differences are small. While the importance of such differences is application‐dependent, these results indicate broad similarities across methods, particularly for the more data rich Birmingham sites.

**TABLE 3:** Linear Regression Results for Daily Maximum WBGT in Downtown Birmingham, Suburban Birmingham, and Wilcox County

**Downtown Birmingham**	Mean	*R* ^2^			RMSD		
Method		NLDAS	WS/iButton	NLDAS/iButton	NLDAS	WS/iButton	NLDAS/iButton
WS	24.94	0.94	0.96	0.95	0.82	1.17	1.13
NLDAS	25.25		0.90	0.89		1.16	1.13
WS/iButton	25.91			0.99			0.26
NLDAS/iButton	25.81						

**Suburban Birmingham**	Mean	*R* ^2^			RMSD		
Method		NLDAS	WS/iButton	NLDAS/iButton	NLDAS	WS/iButton	NLDAS/iButton
WS	24.76	0.95	0.97	0.96	0.77	0.92	0.77
NLDAS	24.90		0.93	0.92		1.03	1.02
WS/iButton	25.48			0.98			1.13
NLDAS/iButton	24.47						

**Wilcox County**	Mean	*R* ^2^			RMSD		
Method		NLDAS	WS/iButton	NLDAS/iButton	NLDAS	WS/iButton	NLDAS/iButton
WS	28.42	0.10	0.62	0.58	2.03	1.26	1.28
NLDAS	28.00		0.32	0.28		1.52	1.58
WS/iButton	28.18			0.96			0.57
NLDAS/iButton	28.62						

### 3.5 Occupational Heat Stress Thresholds

**FIGURE 9:**
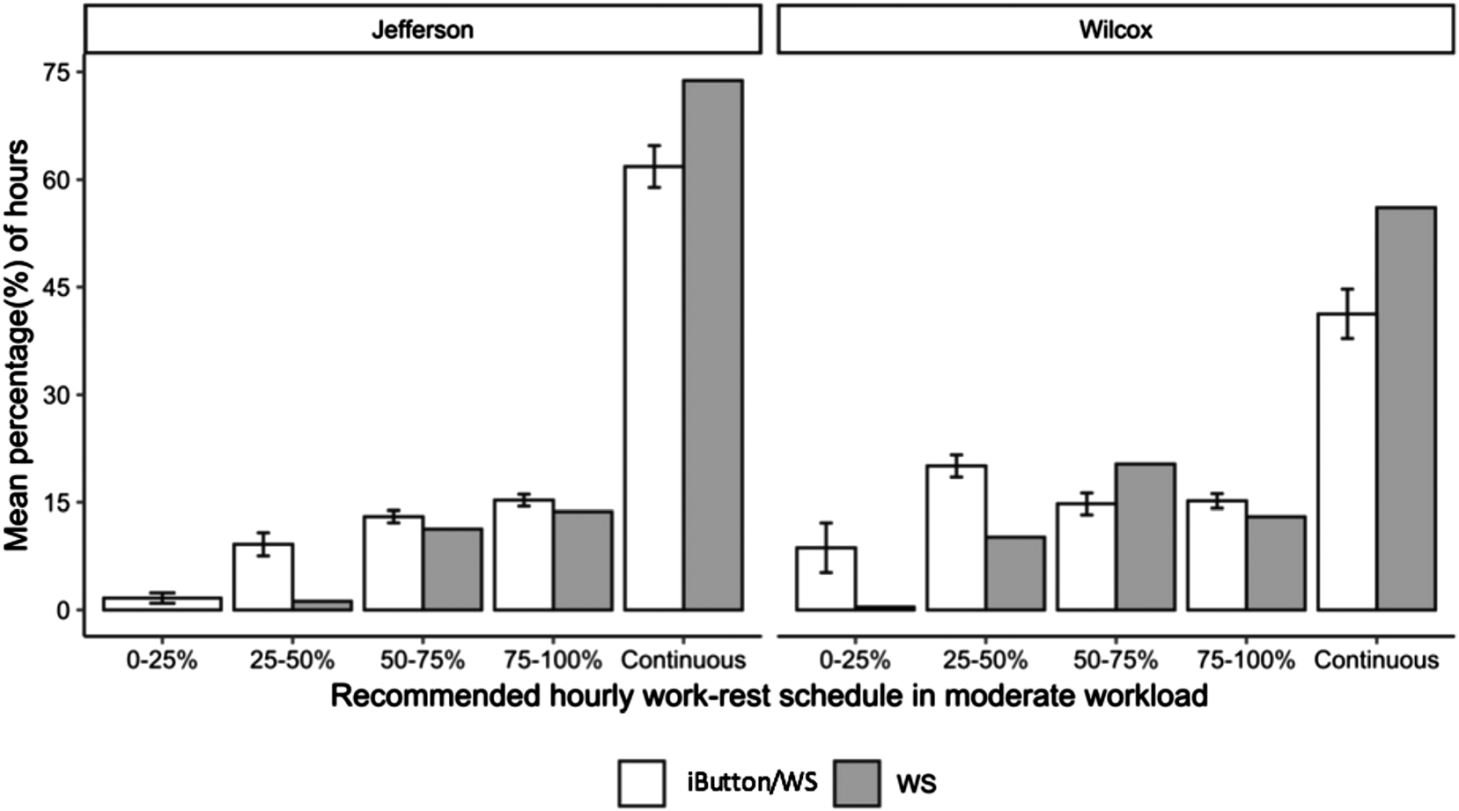
Mean percentage of hours in recommended work‐rest schedule based on WBGT index across all iButtons (mean and 95% confidence interval) in moderate workload. Moderate workload has a metabolic rate of approximately 300 W, examples are normal walking and moderate lifting. Thresholds for % work recommended per hour: Continuous work <28°C, 75–100% work (45 to 60 min work/0 to 15 min rest per hour) >28°C and <29°C, 50–75% work (30 to 45 min work/15 to 30 min rest per hour) >29°C and <30°C, 25–50% work (15 to 30 min work/30 to 45 min rest per hour) >30°C and <31.5°C, 0–25% work (0 to 15 min work/45 to 60 min rest per hour) ≥31.5°C (NIOSH, 2017). *n* = 28 iButtons in Jefferson County and *n* = 14 iButtons in Wilcox County. Only one WS in each county.

As previously discussed, heat stress is a health concern in many work environments. Therefore, employers should follow work‐rest guidelines to ensure employees are taking appropriate rest periods when working in environments that increase the risk of heat‐related illness. The NIOSH has issued recommended heat stress exposure limits and appropriate work‐rest periods, for heat acclimatized workers (NIOSH, 2017). To assess our WBGT estimates in terms of these guidelines, work‐rest periods for a worker with a moderate workload (300 Watts [W] metabolic heat based on ACGIH “2017 TLVs and BEIs” Table 3) are presented in Figure 9 (ACGIH, 2017). The purpose of this analysis is to assess the adequacy of easily obtained remote WBGT estimates (in this case, a central WS estimate) for monitoring local WBGT conditions relevant to worker safety. Or, stated differently, whether using a low cost local temperature and humidity measurement device like an iButton has any value for heat risk monitoring relative to the WS estimate.

On average, 61.8% ± 1.5% (mean ± standard error [SE]) fell in the continuous work category by using measurements with iButtons, while 73.8% of hours fell into the same category by using WS alone in Jefferson County. In Wilcox County, 41.3% ± 1.7% fell into the continuous work‐rest category by using measurements with iButtons, while 56.1% of hours fell into the same category by using WS alone. When we look at the higher risk categories, a significantly higher percentage of work hours fell into the 0–25% work, 25–50% work, 50–75% work, and 75–100% work categories using the iButtons vs. WS alone in Jefferson County. Results in Wilcox County were similar except in the 50–75%, where iButton data estimated 14.8% ± 0.8% hr vs. WS estimated 20.3% hr. Overall, results show WS data alone estimated significantly more hours not requiring rest to compensate for heat exposure. This suggests that using WS data alone to recommend work‐rest schedules may place workers at a higher risk of overexposure to heat due to longer work time and shorter rest time per hour. These results are particularly interesting when we consider losses in work capacity and labor productivity due to heat stress. For example, Kjellstrom et al. (2018) has estimated substantial losses by the end of the century, mostly due to impacts in the southeastern United States, where heat stress already limits work capacity.

### 3.6 iButton and NLDAS Predictions

**FIGURE 10:**
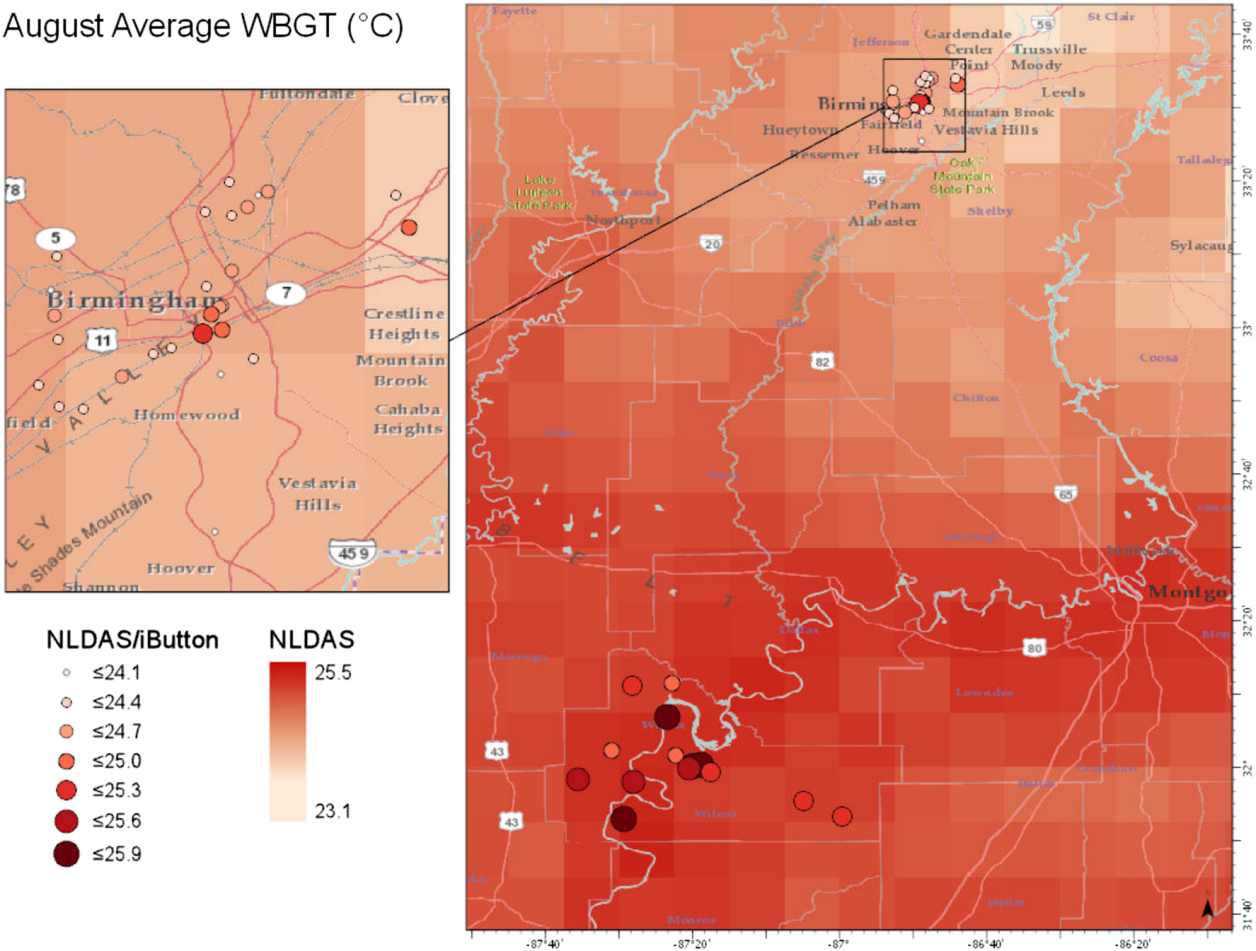
Map of central Alabama showing NLDAS (grid) and NLDAS/iButton (points) calculated WBGT (°C) in August of 2017.

While NLDAS‐informed WBGT estimates were not always as accurate as those that used WS data, their performance was comparable by most metrics at the Birmingham site, and it is likely that Wilcox County performance would improve for a longer analysis period. Methods that use NLDAS have the important advantage of being applicable across space and time, using NLDAS gridded fields. As a demonstration of this capability, we apply our methods to estimate WBGT in new locations, where a Kestrel monitor had not been installed. iButton and NLDAS data were used for this analysis, and we make use of a larger population of iButtons (*n* = 42) that were deployed during this period of analysis as part of the ENACT study. The opportunity allowed us to show (1) how calculations of WBGT differ using NLDAS vs. NLDAS/iButton and (2) how WBGT changes across central Alabama. To do this, NLDAS data were downloaded based on coordinates from all 42 iButtons and combined with iButton measurements to calculate NLDAS/iButton WBGT at each location. Time‐averaged NLDAS GEOTIFF files of central Alabama were also downloaded and used to calculate a gridded map of NLDAS WBGT. These two files were overlain to show the estimates for NLDAS and NLDAS/iButton WBGT for the month of August 2017 (Figure 10).

Summary statistics show that NLDAS vs. NLDAS/iButton are not very highly correlated (*R* ^2^ = 0.67). The average bias is −0.48, RMSE = 0.60, and regression coefficient = 0.92. The low *R* ^2^ value indicates that iButton data have a large impact on WBGT estimates. Also, the NLDAS gridded cells are somewhat large and are therefore not very sensitive to potential local variation in WBGT. The spatial resolution of each method can be seen in the magnified map of Birmingham, which shows that the NLDAS grid does not predict small changes in WBGT seen in the NLDAS/iButton estimates (Figure 10). The results also show that for August 2017, using NLDAS/iButton data generally predicted higher WBGT values than just using NLDAS.

We must also refer to Table 2 in order to interpret results. Based on the NLDAS *R* ^2^ values of downtown and suburban Birmingham (*R* ^2^ > 0.90), and considering the fact that Wilcox had a much smaller sample size, we believe that NLDAS data are a fairly adequate source for calculating WBGT. That being said, we also see that, in all cases, when iButton data are added to NLDAS the *R* ^2^ increases (Table 2). Therefore, we expect that by combining NLDAS and iButton data we are increasing the accuracy of our predictions. This would make the NLDAS/iButton coordinate points a better estimate for WBGT values in those areas. Although iButton data better predict WBGT, the NLDAS grid is still useful because it shows general patterns in WBGT data without referring to the actual temperature values. From this figure, we see that heat stress is greater towards the more southern parts of the state (Figure 10). This application is just one example of how our results can be used to make predictions about heat stress. The question of whether the increase in accuracy afforded by adding iButton data to NLDAS‐based WBGT estimates is worth the effort of installing iButtons will depend on application. From the perspective of general climate hazard mapping or comparisons across large regions, NLDAS could well be sufficient. For application to detailed health studies or to establishing safe work standards (as described in section 3.5), the use of iButtons could be valuable for improving accuracy of local WBGT estimates while avoiding the expense of installing multiple WBGT sensors. We do note that the advantage of applying local measurements only applies to the environment in which the measurement device is installed. An iButton (or WS) placed in an open field will yield estimates representative of that open field and not, for example, of more sheltered or shaded neighboring areas.

## 4 Conclusions

Heat stress is a significant health risk, and WBGT offers an established metric for estimating heat stress risks in indoor and outdoor environments. As the calculation of WBGT depends on nonstandard meteorological measurements that are often unavailable, there is value in developing proxy methods that estimate WBGT from more readily available observations or models. This study is motivated by the need to compare WBGT estimates derived using a range of publicly available or low cost data sources to increase the availability of WBGT estimates for health applications. The evaluation of multiple data sources for WBGT estimation is, to our knowledge, a novel contribution of this study.

Based on our results, we believe that all of our proxy methods are acceptable for estimating WBGT in downtown and suburban Birmingham for general risk mapping applications. In both locations, we observed slight variations in correlations, but the Fisher's r to z transformation showed that there were no statistically significant differences (Table 2). However, the addition of local iButton data to either WS or NLDAS estimates led to a decrease in RMSE (*p* < 0.05) and bias (*p* < 0.05) in WBGT estimates, relative to estimates that did not benefit from site‐specific measurements. These differences indicate that measurements taken in close proximity to the study site will likely increase accuracy of predictions. In Wilcox County, it is more difficult to draw conclusions because of the small sample sizes. There are statistically significant differences between methods, and our available results indicate that the WSs offer an acceptable method for calculating WBGT. However, more data are needed to make definitive recommendations about potential data sources for this site. We also note, again, that the Kestrel instrument employs a combination of measurement and calculation to estimate WBGT, and that it is prone to measurement error like any instrument. Hence, evaluation of proxy methods against the Kestrel readings could be influenced by errors in both the proxy methods and the Kestrel device.

Looking forward, it would be valuable to collect WBGT measurements from a wider range of locations in order to better understand the accuracy and generalizability of these WBGT estimation methods beyond the ENACT study's central Alabama focus region. A robust WBGT estimation method, grounded in measurements obtained across diverse environments, could be a powerful tool for studies and interventions related to heat stress and its health impacts.

## Conflict of Interest

The authors declare no conflicts of interest relevant to this study.
